# State-Level Consumer Protection Policies and Medigap Premiums and Plan Offerings

**DOI:** 10.1001/jamahealthforum.2025.0708

**Published:** 2025-04-25

**Authors:** Angela Liu, Melinda Buntin, Gerard Anderson

**Affiliations:** 1 Department of Health Policy and Management, Johns Hopkins Bloomberg School of Public Health, Baltimore, Maryland; 2 Carey School of Business, Johns Hopkins University, Washington, DC; 3 Senior Associate Editor, *JAMA Health Forum*; 4 Division of General Internal Medicine, Johns Hopkins Medicine, Baltimore, Maryland

## Abstract

This cross-sectional study describes the number of Medigap plans offered, average Medigap premium, and percentage of Medigap enrollment.

## Introduction

Traditional Medicare supplemental insurance, Medigap, provides additional financial protections for beneficiaries enrolled in traditional Medicare. Beyond an initial 6-month period during which a beneficiary first ages into Medicare, in 42 states and Washington, DC, insurers use medical underwriting to set the price of premiums based on the beneficiary’s age and health status (individual rating). Eight states have passed Medigap consumer protection policies, such as requiring insurers to sell Medigap plans to anyone applying (guaranteed issue) and to offer 1 rate for everyone applying (community rating). Beneficiaries with high expected medical costs may have difficulty affording the individually rated premiums, and several states, including California, Hawaii, Pennsylvania, and South Carolina, are currently considering reforms that require guaranteed issue and community rating.^1,2^ The concern is that if such reforms are enacted, insurers will increase premiums or exit the market due to adverse selection, potentially affecting Medigap market stability^3^ and beneficiary access.^4^ This analysis examines this trade-off by describing the number of Medigap plans offered, average Medigap premium, and percentage of Medigap enrollment by state-level Medigap consumer protection policies.

## Methods

This study followed the Strengthening the Reporting of Observational Studies in Epidemiology (STROBE) reporting guidelines for cross-sectional studies. Institutional review board approval was not sought because no human participants were involved. We used the Mark Farrah Associates Medicare Supplemental Market Dataset, which includes Medigap plan enrollment, premiums, claims, and loss ratios nationwide.^5^ We examined Medigap plan G, the most generous and popular plan that was offered in 2023, and excluded plans with no enrollees (n = 142) and plans operating in Wisconsin, Massachusetts, and Minnesota, as Medigap plan structures in these states deviate from federal guidelines.

States were classified into 1 of 3 mutually exclusive groups: no Medigap protections (40 states and Washington, DC), community rating only (Washington, Vermont, and Arkansas), and community rating plus guaranteed issue (Maine, New York, and Connecticut). We supplemented these data with publicly available 2023 Medicare enrollment data provided by the US Centers for Medicare & Medicaid Services, including state-level Medicare enrollment and county-level benchmark spending.^6^

Summary statistics were calculated for the number of Medigap offerings per Medicare capita, percentage of traditional Medicare beneficiaries enrolled in Medigap, unadjusted premiums, and premiums adjusted for benchmark spending to account for differences in payment rates and use patterns across states. Premium adjustment required computing state-level averages of traditional Medicare spending weighted by county-level traditional Medicare population, determining each state’s weighted average compared with the national average, and scaling state-level Medigap premiums accordingly. All analyses were performed using R, version 4.3.3 (R Foundation).

## Results

Across the 47 states and Washington, DC, the population-standardized mean number of Medigap plan G plans available was 15.7 per 100 000 Medicare enrollees (Figure). The number of plans offered per 100 000 beneficiaries ranged from 17.3 for states with no Medigap consumer protection policies to 4.1 for states with community rating and guaranteed issue. Similarly, population-adjusted enrollment ranged from 14.6% in states with no protections to 2.4% in states with community rating and guaranteed issue. The mean annual adjusted Medigap premium was $2497 for states with no Medigap consumer protection policies, $2290 for states with community rating only, and $2760 for states with guaranteed issue and community rating (Table).

**Figure.**
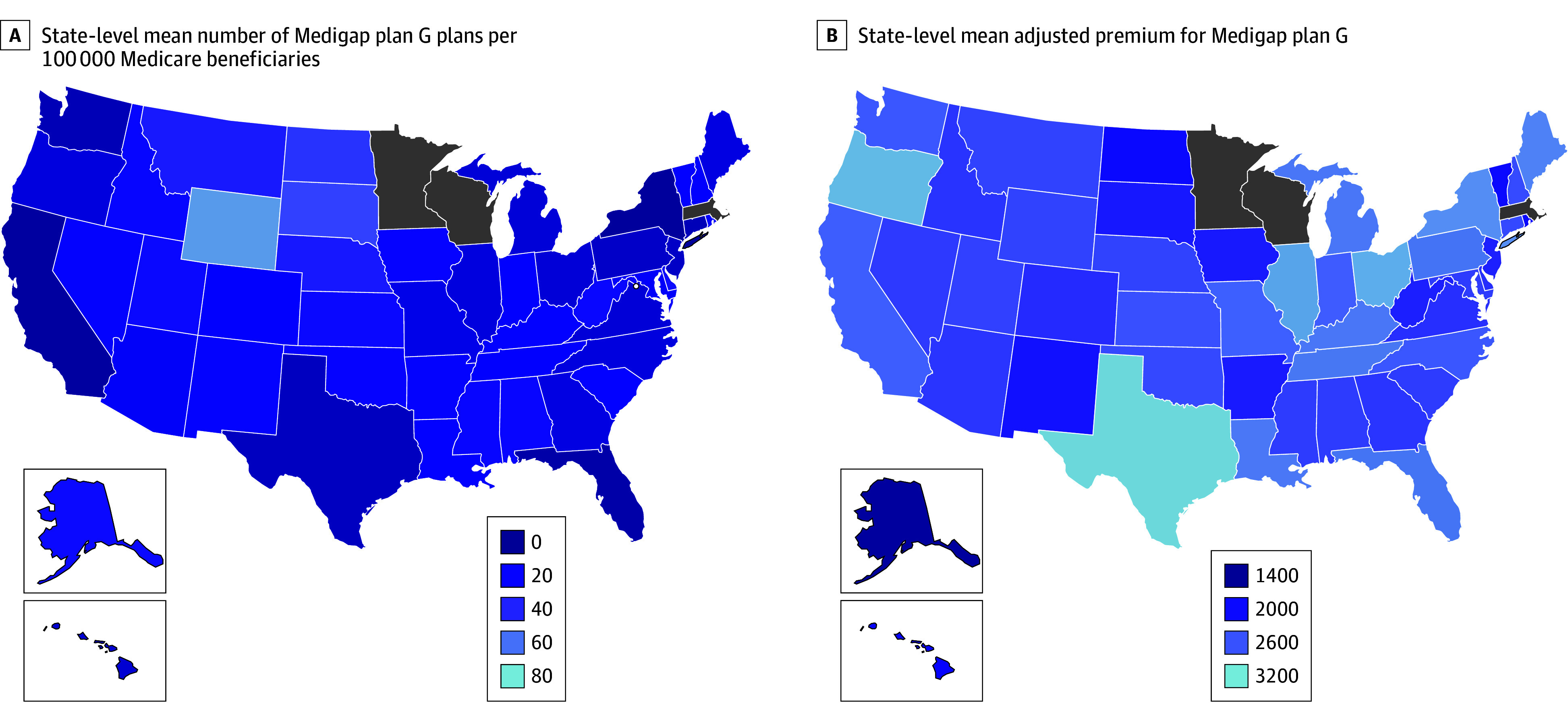
State-Level Mean Number of Medigap Plan G Plans per 100 000 Medicare Beneficiaries and State-Level Mean Adjusted Premiums for Medigap Plan G

**Table.** Number of Plans (Population Adjusted), Premiums, and Percentage Enrolled by State-Level Medigap Consumer Protection Policy Groups for Medigap Plan G Plans

Characteristic	No. of plans per 100 000 Medicare beneficiaries		Unadjusted premiums, $		Adjusted premiums, $		Population-adjusted Medigap enrollment, %
	Mean (SD)	Min to max (IQR)	Mean (SD)	Min to max (IQR)	Mean (SD)	Min to max (IQR)	
No protections	17.3 (13.8)	1.0-67.6 (15.2)	2500 (344)	1516-3253 (352)	2497 (329)	1420-3135 (280)	14.6
Community rating only	11.3 (6.9)	3.4-16.3 (6.5)	2238 (330)	1910-2570 (330)	2290 (316)	2001-2628 (313)	9.9
Guaranteed issue and community rating	4.1 (3.7)	0.6-8 (3.7)	2823 (247)	2623-3100 (238)	2760 (172)	2564-2887 (162)	2.4

Abbreviations: max, maximum; min, minimum.

## Discussion

In this cross-sectional study, in states with community rating and guaranteed issue, the Medigap market persisted, although insurers offer fewer Medigap plan G plans and set higher premiums. In these states, all beneficiaries were subject to higher premiums, regardless of their health status, which distributed the financial burden of health insurance across a larger population. Lower Medigap enrollment in community rating and guaranteed issue states could have been due to fewer plans with higher premiums resulting in lower uptake or beneficiaries waiting to enroll until they experienced illness. This work was limited by its cross-sectional design. As states consider Medigap reform, future work should examine the effects of changes in Medigap market policies.

## Notes

Supplement. Data sharing statement
