# Supplementary material for: State-Level Consumer Protection Policies and Medigap Premiums and Plan Offerings
Source: JAMA Health Forum. 2025 Apr 25;6(4):e250708. doi: 10.1001/jamahealthforum.2025.0708 (PMC12032563; doi:10.1001/jamahealthforum.2025.0708)
Supplement: Supplement. — Data sharing statement [file jamahealthforum-e250708-s001.pdf]

Data Sharing Statement

Liu. State-Level Consumer Protection Policies and Medigap Premiums and Plan Offerings. *JAMA Health Forum*. Published April 25, 2025. doi:10.1001/jamahealthforum.2025.0708

Data
Data available: No
